# Evaluation of a web-based ECG-interpretation programme for undergraduate medical students

**DOI:** 10.1186/1472-6920-8-25

**Published:** 2008-04-23

**Authors:** Mikael Nilsson, Gunilla Bolinder, Claes Held, Bo-Lennart Johansson, Uno Fors, Jan Östergren

**Affiliations:** 1 Department of Medicine, Karolinska University Hospital, Solna, 171 76, Stockholm, Sweden; 2 Department of Cardiology, Karolinska University Hospital, Solna, 171 76, Stockholm, Sweden; 3 Department of Clinical Physiology, Karolinska University Hospital, Solna, 171 76, Stockholm, Sweden; 4 Department of Learning, Informatics, Management and Ethics, Karolinska Institutet, 171 77, Stockholm, Sweden

## Abstract

**Background:**

Most clinicians and teachers agree that knowledge about ECG is of importance in the medical curriculum. Students at Karolinska Institutet have asked for more training in ECG-interpretation during their undergraduate studies. Clinical tutors, however, have difficulties in meeting these demands due to shortage of time. Thus, alternative ways to learn and practice ECG-interpretation are needed. Education offered via the Internet is readily available, geographically independent and flexible. Furthermore, the quality of education may increase and become more effective through a superior educational approach, improved visualization and interactivity.

**Methods:**

A Web-based comprehensive ECG-interpretation programme has been evaluated. Medical students from the sixth semester were given an optional opportunity to access the programme from the start of their course. Usage logs and an initial evaluation survey were obtained from each student. A diagnostic test was performed in order to assess the effect on skills in ECG interpretation. Students from the corresponding course, at another teaching hospital and without access to the ECG-programme but with conventional teaching of ECG served as a control group.

**Results:**

20 of the 32 students in the intervention group had tested the programme after 2 months. On a five-graded scale (1- bad to 5 – very good) they ranked the utility of a web-based programme for this purpose as 4.1 and the quality of the programme software as 3.9. At the diagnostic test (maximal points 16) by the end of the 5-month course at the 6th semester the mean result for the students in the intervention group was 9.7 compared with 8.1 for the control group (p = 0.03).

**Conclusion:**

Students ranked the Web-based ECG-interpretation programme as a useful instrument to learn ECG. Furthermore, Internet-delivered education may be more effective than traditional teaching methods due to greater immediacy, improved visualisation and interactivity.

## Background

Worldwide, cardiovascular disease is estimated to be the leading cause of death in the world 2020 [1]. Electrocardiography (ECG) continues to be the most commonly used laboratory procedure for the diagnosis of heart disease. Introduced in 1902 by Einthoven [2], the procedure reflects electrical changes associated with primary or secondary myocardial processes for example coronary artery disease, hypertension, and electrolyte abnormalities [3].

Physicians in most clinical specialities, including general practice are expected to have a sufficient knowledge about ECG interpretation to be able to make accurate diagnoses, decide on patient management or further referrals. An adequate knowledge base should include the ability to define, recognize, and understand the basic pathophysiology of certain electrocardiographic abnormalities [3]. Our experience, together with existing research, suggests that most medical students do not feel competent in their interpretation of ECG [4]. In the extension this could negatively influence patient management decisions and could threaten patient safety [5,6].

Despite the importance of ECG-training, clinical tutors seem to have increasing difficulties in meeting the need for more training in ECG interpretation due to shortage of time or other reasons. Thus, alternative and flexible ways to learn and practice ECG interpretation are needed [7].

Education offered via the Internet is usually readily available, geographically independent and flexible [8]. If designed in a proper way with a good pedagogical method, such as active learning, simulation and with a case-based approach, interactive learning systems may improve the quality of education, facilitate visualisation and understanding and also increase the effectiveness of the education [9-12].

The aim of this project was to evaluate a web-based comprehensive ECG interpretation programme for medical students at Karolinska Institutet during the internal medicine course. We assessed the attitude of the students towards web-based ECG learning in general and the usefulness of a specific programme. We also intended to objectively assess the impact and effectiveness of web-based ECG tutorials by comparing the learning outcomes in the intervention and the control group with a diagnostic test.

## Methods

### The ECG-training programme

Clinical ECG interpretation requires basic knowledge from several fields of medicine, such as anatomy, physiology and clinical pathophysiology. A web-based ECG interpretation programme "EKGtolkning.com" was selected for this study. EKGtolkning.com has previously been utilized in the continuing medical education (CME) of clinically active physicians in an ECG interpretation course at Karolinska Institutet The program is also used in an online course for nurses. The programme was modified with a pre-test module to suit the needs of undergraduate medical education in terms of test situations. The programme was designed to serve both as a complement to the standard ECG education, or to be used as "stand-alone" tool for self-regulated learning.

The web-based system contains all information that medical students at Karolinska Institutet should need during a basic ECG-interpretation course [3,7]. The content is divided into separated parts: Clinical introduction, The ECG in detail, the pathological ECG and clinical ECG cases.

• *Clinical introduction *gives a basic understanding of the electric activity of the heart and how this is registered using electrocardiography. It also includes clinical symptoms, anatomy and physiology to give a basic understanding of the placement and orientation of the heart within the chest and its relation to the ECG-electrodes. This section also covers the anatomy and function of the cardiac conduction system as well as the electrophysiological properties of the heart.

• The *ECG in detail *covers the basic principles of ECG, where the ECG registration is discussed in detail. A summary with conclusions and a thorough review of the ECG-interpretation checklist is included.

• The section with *pathological ECG:s*, covers common pathological ECG anomalies. 25 conditions are presented and explained.

• The *clinical ECG cases *consist of 70 specially selected ECG:s, together with short descriptions of the clinical situations in which the ECG-tracings were recorded. They exemplify a number of typical cases, in which ECG interpretation is important.

The programme was designed in accordance with parts of several learning theories, among others Kolb's Experiential Learning [13]. The theory includes four elements: concrete experience, observation and reflection, the formation of abstract concepts and testing in new situations.

**Figure 1:**
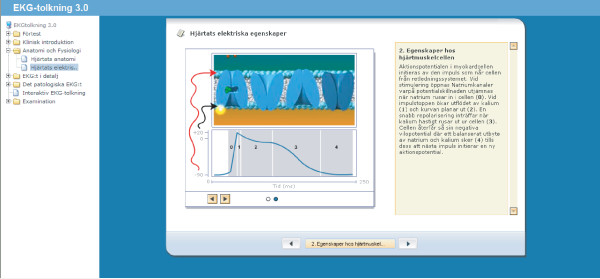
The learning object explains the depolarization process in the myocardium by an animation and an explanation in text.

**Figure 2:**
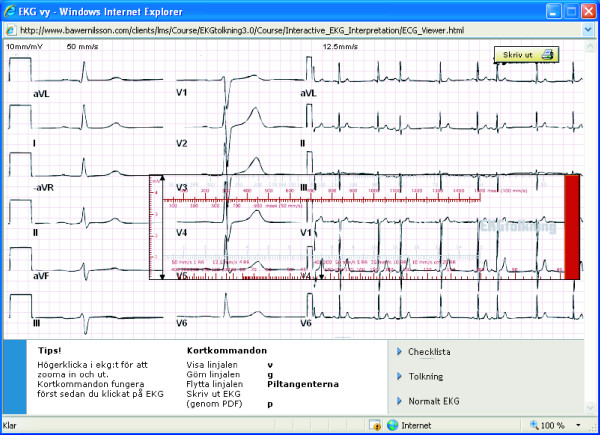
The learning object presents one of the ECG cases in the interactive ECG interpretation module. The cases are based on a clinical history. The student is asked to interpret the ECG. One of the tools they can use for this purpose is the special moveable ruler. The ECG can be magnified together with the ruler for measuring the components of the ECG-complex. The student's interpretation can be compared with the interpretation of an expert.

**Figure 3:**
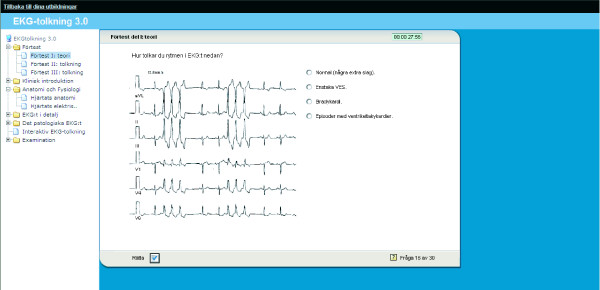
The learning object is an example of one of the theoretical questions. The students are asked to interpret the rhythm by choosing from one of 4 alternatives. Directly after answering the question feedback is given, where the right answer is enlightened.

The Programme uses three pedagogical and technical solutions to implement the different parts of the course:

1. The core learning objects (Fig 1) constitute the theoretical backbone of the course. The core learning objects is made by text, animations and illustrations.

2. The interactive ECG interpretation module (Fig 2) allows students to interpret authentic ECG registrations using a number of tools and utilities. Clinical history and information on the actual ECG is also presented. The module presents an opportunity to practice, apply and develop interpretation skills based on the assumption that the students interpretation combined with the possibility to compare with an interpretation made by an expert will facilitate the gain of knowledge. The module thus tries to mimic the clinical reality in order to optimize ECG understanding and interpretation [14,15].

3. The linear test system (Fig 3) is a tool for running and tracking tests. The questions are defined in an external file and interpreted by the system. The questions are constructed in order to achieve maximal learning levels according to Blooms Taxonomy of Educational Objectives [16].

### Test setting

Medical students from the sixth semester at the Karolinska Institutet who attended the internal medicine course at Karolinska University Hospital (12 males, 20 females, mean age 27 years) were offered to use the programme from the start of their course as an adjunct to the conventional training in ECG interpretation. The ECG system was introduced during a 40 minute lecture and students were encouraged to use the programme for training on a voluntary basis. The students was informed about the study and gave consent to it as well as to publication. It was possible to use the system at any time during the course through any Internet connected computer. Logged on, the students had access to all material in the program without demands of sequence or test of skills.

User activity was automatically logged. An evaluation of attitudes towards web-based ECG-learning in general and the utility of the specific programme was obtained after 2 months. This was performed as part of an individual semi-structured interview with all students during the course of an individual personal development dialogue, by one of the authors (JÖ) asking about the students opinion of the a) general utility of web based ECG as a learning tool and b) the quality of the specific programme used. The opinions were ranked on a 5-graded scale.

A diagnostic test was performed at the end of the 5-month course to assess the possible effect on skills in ECG interpretation/reading. Students from the corresponding sixth semester course, at another teaching hospital in the Stockholm area (14 males, 16 females, mean age 26 years), without access to the web-based programme served as a control group. However, the latter group of students received 3.5 days of extra training in clinical physiology, including interpretation of ECG. In addition both the intervention group and the control group had received a 15 hour long course in ECG-interpretation during the 5^th ^semester.

Grading of the individual diagnostic test results was performed without the examiner knowing the identity of the subjects or to which course they belonged. Results of the test were compared between the two courses by Students T-test (two-sided).

## Results

20 of the 32 students in the test group course volunteered to use the ECG-system. In the control group, 30 students fulfilled the course without having access to the ECG program.

At the individual semi-structured interview the 20 students in the test group who had used the programme were asked to rank their apprehension of the system based on a five-graded scale (1- bad to 5 – very good). In average, they ranked the utility of the web-based programme for this purpose as 4.1 (SD 1.0) and the quality of the programme software as 3.9 (SD 0.3).

**Figure 4:**
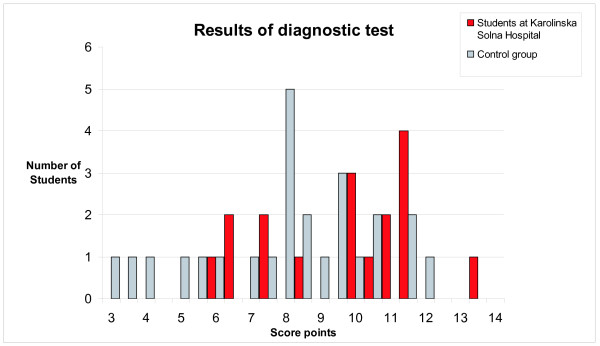
Individual scores at the diagnostic test by the end of the sixth semester. Students in the test group are represented in red bars (n = 17) and in the control group in grey bars (n = 25). Maximal points were 16. The difference between the groups was statistically significant (p = 0.03).

The diagnostic test consisted of interpretation/reading of 8 different ECG:s. These ECG:s had previously been used in the examination by the same two groups of students during their 5th semester At the optional diagnostic test by the end of the sixth semester, 17/20 students in the test group, and 25/30 in the control group completed the voluntary test. The test group scored significantly better (average 9.7 (SD 2.19)) as compared with 8.1 (SD 2.47) for the control group, (p = 0.03). Maximum points were 16. The individual scores at the test are depicted in Figure 4.

## Discussion

The importance of a basic understanding of ECG interpretation is of critical importance for physicians but despite this gaps in knowledge exist. For example, in a recent experimental study, it was shown that the performance of internal medicine and emergency medicine residents in ECG-interpretation was rather low [17]. The competency in ECG-interpretation improves with training [18], but training seems to be deficient at many universities. Thus, it is important to find better ways for medical students to begin and maintain this training.

The students indicated a positive attitude to web-based ECG-learning in general and the utility of the specific programme and the web-based ECG interpretation programme used in this study seemed to increase skills in ECG-interpretation when evaluated by the diagnostic test. Our results thus suggest benefits with added web-based ECG-learning programme over only conventional teaching of ECG. However a limitation with this study is that some of the students did not perform the voluntary diagnostic test and that a pre-test was not performed. There is also a possibility of a volunteer bias in these kind of studies, a fact that is always existing when there are few opportunities to make the test compulsory before a new educational tool is tested. Additional studies are thus needed to assess the possible advantages with web-based ECG-learning and if it can replace conventional learning. To investigate learning outcomes in more detail and also to assess if these outcomes are related to background factors such as time spent in the programme and individual learning techniques we are projecting further studies.

Web-based education is a branch of Electronic learning (E-learning) that is increasingly used for medical education. The major reason for this seems to be the general advantages of Internet like availability, geographic independence, flexibility, improved visualization and the opportunity of interaction. Hudson [9] points out the fact that most studies in medical E-learning compare traditional education with e-learning without evaluation of the "*if, what and why*" of possible pedagogical benefits of E-learning. In our case, students reported some specific benefits of the ECG tool like the possibility to use it individually and at any time.

Web-based educations might have a few different pedagogical and technical solutions. In this web-based ECG learning system we used three specific types of pedagogical and technical approaches:

1. The theoretical basis of ECG was explained through text, animations and images.

2. The self-evaluation system. Most students like to test their own knowledge and skills. In this system, they can use the built-in test before they go to the real exam in the course.

3. The interactive ECG interpretation module, which allows students to practise interpretation of authentic ECG registrations using typical tools and utilities. This module presents an opportunity to practise, apply and develop interpretation skills.

Even though the pedagogical and technical solutions used in this system seems to be of value to improve learning outcomes, a more thorough evaluation is needed to find solutions applicable for Web-based education in general.

For example, we showed 32 students at one single occasion how to use the web-based ECG interpretation programme in the beginning of the period. 62% of the students voluntarily used the programme. In our opinion this is a fairly good proportion of students using the system and others have had similar numbers of student participation introducing e-learning [19].

In this study we did not investigate why some students used the programme and others did not. In some cases the reason might be due to lack of computers, or a behavioural resistance [20] to a relatively new way of studying. indicating the opposite. It may be argued that the better result in our intervention group may be due to a positive selection of students but Hahne et al did not show that students who participate or agree to use e-learning are better students than those who do not use e-learning [19]. Further scrutinizing of the students readiness to use or not use new educational instruments is important in faculty development.

Optimal methods to learn and maintain ECG interpretation skills and competence are not yet fully understood [21,22]. The different kinds of ECG:s that a physician in general practice needs to recognize is well known, but the number of ECG-examples needed to be interpreted to learn and maintain ECG competence is still a question. In the Clinical Competence Statement on Electrocardiography from the American College of Cardiology and the American Heart Association [3] a total number of 500 ECG:s is demanded to learn ECG-interpretation and a 100 ECG:s yearly to maintain ECG interpretation skills. These indicated numbers are based more on opinion than statistical surveys.

## Conclusion

In conclusion, the described ECG training system seems to have a good potential to facilitate ECG learning both in individual self-learning and to improve the effectiveness of teacher-controlled tuition. Due to the lack of pre-intervention testing and possible volunteer bias in our study more research need to be performed to fully understand the "*if's, where's and how's*" of using computer based ECG training. However this interactive ECG interpretation system together with a learning management system might be a suitable model to be used to further study this subject.

## Competing interests

MN is a shareholder in the company which has developed the web-based ECG-interpretation programme. There are no other conflicts of interest in relation to this manuscript.

## Authors' contributions

MN together with co-worker invented and developed the program structure and put together the various sources of data into a comprehensive system, MN wrote the manuscript with the help of GB and JO who also participated in the design of the study and the evaluation of data, CH and BLJ were responsible for the students ECG educational program and the introduction of the web-program and UF participated in design, pedagogical counselling, evaluation and interpretation of data. All authors read and approved the final manuscript.

## Pre-publication history

The pre-publication history for this paper can be accessed here:


